# The processing, preparation, and cooking practices of small fish among poor Ghanaian households: An exploratory qualitative study

**DOI:** 10.1007/s40152-023-00300-w

**Published:** 2023-04-12

**Authors:** Yaw Opoku Agyei-Mensah, Theophilus Annan, Ragnhild Overå, Amy Atter, Anne Hatløy, Peter Andersen, Kojo Odei Obiri, Richard Stephen Ansong, Bhagya Janananda, Matilda Steiner-Asiedu, Marian Kjellevold

**Affiliations:** 1 Centre of International Health, University of Bergen, Bergen, Norway; 2 Council for Scientific and Industrial Research, Food Research Institute, Accra, Ghana; 3 Department of Geography, University of Bergen, Bergen, Norway; 4 Fafo Institute for Labour and Social Research, Oslo, Norway; 5 University of Ghana, Accra, Ghana; 6 Department of Seafood, Nutrition and Environmental State, Institute of Marine Research, Bergen, Norway

**Keywords:** Ghana fishing, Poor household, Small fish, Fish processing, Fish preparation, Fishing community

## Abstract

Small fish are an important part of the diet in Ghana, but malnutrition rates remain high. The nutritional quality of fish consumed in Ghana may be affected by food processing and cooking practices, but the extent to which these processes are practiced among poor Ghanaian households along the coastal belt is unknown. This study explored how poor Ghanaian households process, prepare, and cook meals containing small fish. This exploratory qualitative study used Attride-Stirling thematic network analysis. Respondents were purposively sampled from fishing communities in the coastal regions of Ghana. One-on-one interviews were performed by trained field assistants, audio recorded and videotaped, and transcribed for further data analysis. The most common small fish species identified were anchovies and herrings. Anchovies were fried and eaten whole. Herrings were eaten either smoked or fresh; for fresh herring, the head, fins, and viscera were removed before boiling. Herrings were smoked with the head and viscera; however, both the head and viscera were removed before being added to boiling soup and were not consumed. Anchovies were fried for 10 min, and herrings were boiled for 15–30 min. Processing methods and further meal preparation depend on the small fish species. Nutrient composition and contribution of small fish depend on the processing method, preparation method, and what tissues are eaten. Thus, these results will be of importance for sampling schemes for food composition tables and for the calculation of nutrient intake from small fish.

**Supplementary Information:**

The online version contains supplementary material available at 10.1007/s40152-023-00300-w.

## Introduction

Fish are considered a good source of protein and fatty acids, particularly the long-chain omega-3 polyunsaturated fatty acids eicosapentaenoic acid (EPA) and docosahexaenoic acid (DHA). Additionally, fish provide a variety of micronutrients, including vitamin A, vitamin B12, vitamin D, calcium, iodine, selenium, and zinc (Golden et al. 2021; Reksten et al. 2020; Hicks et al. 2019). Nutrient composition analysis has shown wide variability in the nutritional value of different fish species, with small indigenous fish species consumed whole such as anchovies and herrings being particularly rich sources of iron, zinc, calcium, vitamin B12, vitamin D, and iodine (Hasselberg et al. 2020; Roos et al. 2007).

Heat is used in a variety of ways in fish processing, including boiling, baking, roasting, frying, and grilling (Alipour et al. 2010). It enhances the taste, flavor, and more importantly extends the shelf life, providing year-round availability of fish to consumers (Smida et al. 2014; Oparaku and Nwaka 2013). In addition, smoking improves organoleptic characteristics and reduces the microbial load (Igwegbe et al. 2015; Yusuf et al. 2015).

However, these methods may also have drawbacks. For example, the preparation of fish generates large amounts of byproducts, such as heads, guts, skin, bones, and livers. These byproducts are rich in protein, vitamins, minerals, and essential fatty acids (Smida et al., 2014). Additionally, smoking fish can result in the loss of fats and micronutrients due to fat dripping and increased water loss from the fish, in addition to potentially harmful concentrations of polycyclic aromatic hydrocarbons (PAH). High-temperature smoking modifies protein, lowers the essential amino acids, and may result in the loss of major nutrients (Adeyeye et al. 2019; Kabahenda et al. 2009). Pourshamsian et al. (2012) discovered that frying reduced the levels of EPA and DHA and therefore can affect the proximate composition and fatty acids of fish and fish products. However, smoked fish are still a nutrient-dense food (Hasselberg et al. 2020). Nutrient content varies with species, tissue consumed, and processing method. However, little data are available in the scientific literature on how processing methods or species impact preparation and cooking methods. The present study focused on the food processing and cooking practices of low-income households in the coastal regions of Ghana. Small fish is a staple part of the daily diet in these communities, but the extent to which the nutritional quality of the fish is affected by processing and cooking practices is not well understood. To address this gap in knowledge, the study aims to explore the ways in which small fish is processed, prepared, and cooked by the study participants. Specifically, the study has three aims: (1) to determine which fish species are preferred by the households, (2) to understand which processing methods are preferred for different species, and (3) to determine which parts of the fish are used when cooking a meal.

Globally, the current annual per capita consumption is 20.2 kg (FAO 2022). At present, the annual per capita consumption of fish in Ghana is estimated to be 25 kg, which is higher than the estimated averages of 20.2 kg for the world and 10.5 kg for Africa (FAO 2022). Fish represents about 53.9% of the animal protein consumed in Ghanaian homes (FAO 2020). According to Nti and Lartey (2008), 95% of households in rural Ghana consume small fish daily. Small fish can be defined as all fish below 25 cm in length (Bavinck et al. 2023).

## Materials and methods

**Fig. 1:**
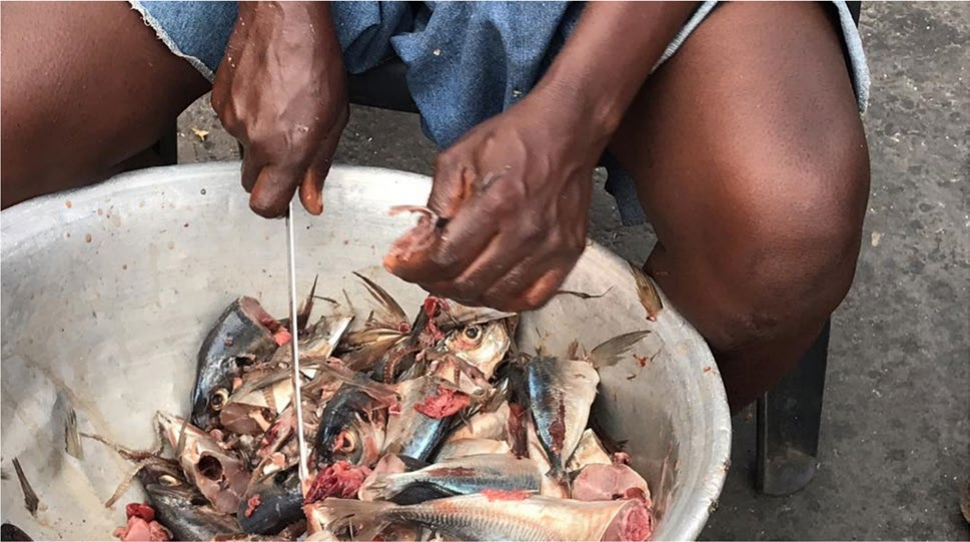
Fish preparation in Ghana

**Fig. 2:**
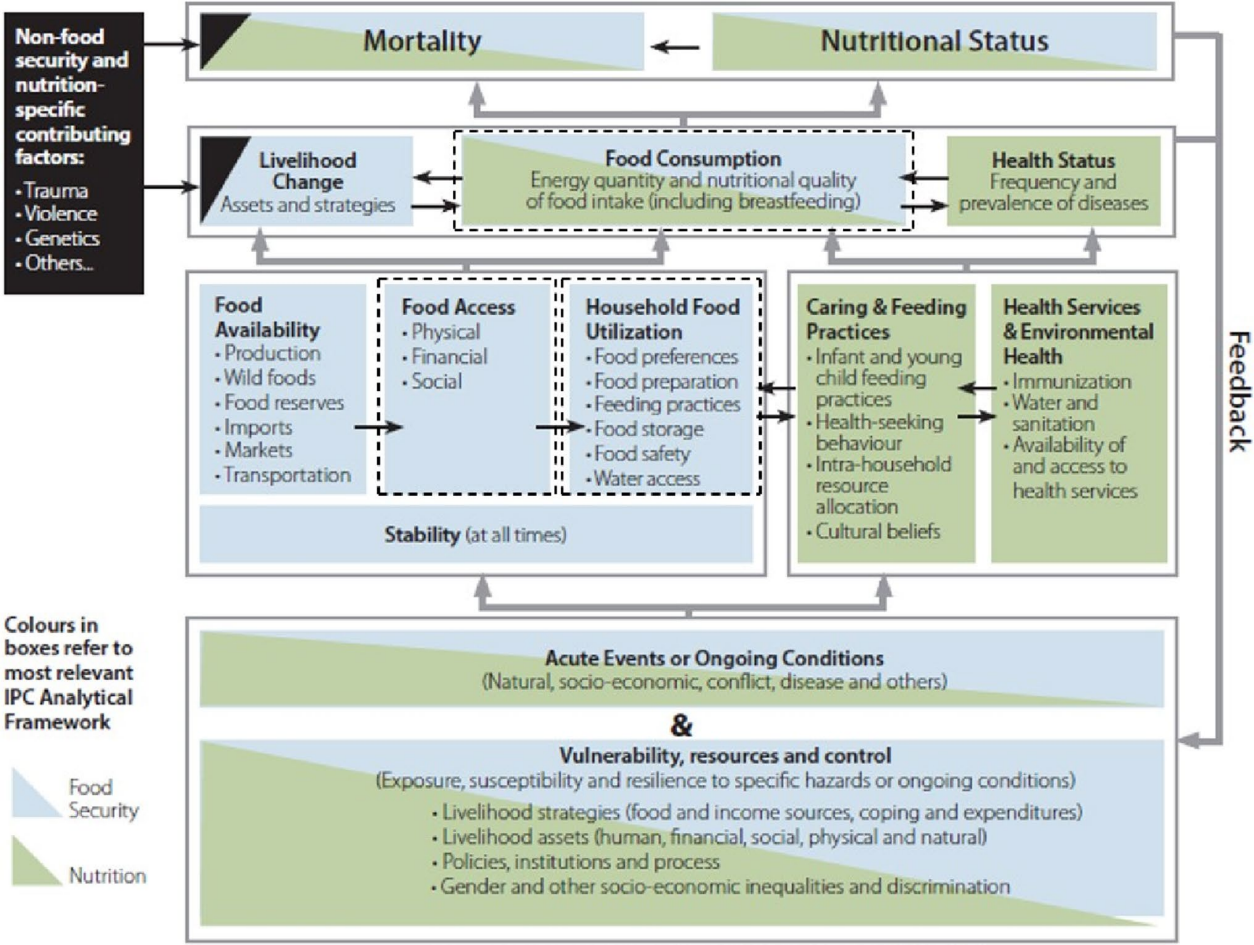
Studied areas of the IPC Integrated Food Security and Nutrition Conceptual Framework adopted from IPC Technical Manual V 3.1 (IPC Global Partners 2021). Highlighted in dark-dashed lines are the relevant areas addressed in this study

The IPC Integrated Food Security and Nutrition Conceptual Framework aims to link food security and nutrition. It considers the interplay between food availability, access, utilization, and stability, as well as the underlying socio-economic and environmental factors that influence these dimensions. The present work addresses “Food Consumption” and “Household Food Utilization” within this Framework (Figs. 1, 2). “Food Consumption” is relevant for food security and nutrition and includes quantity and nutritional quality. The fish species (aim 1), processing method (aim 2), and what parts of the fish are consumed (aim 3) will impact the nutritional quality. “Household Food Utilization” is most relevant for food security and includes, e.g., food preferences, food preparation, feeding practices, and food safety (aims 1–3).

### Study area

**Fig. 3:**
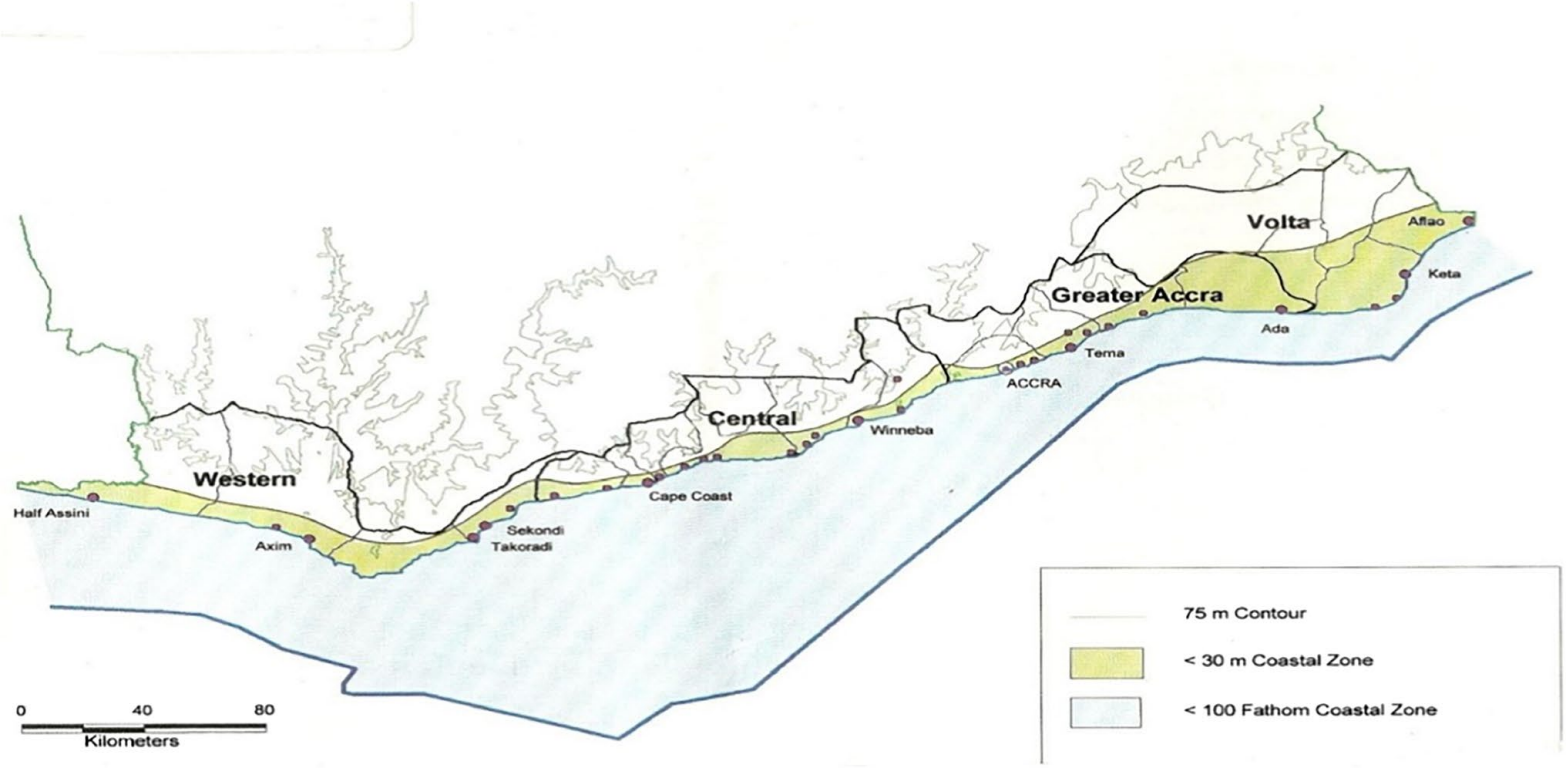
Map of the coastal zone of Ghana (uploaded by Christopher Gordon)

This research was carried out in Ghana’s four administrative coastal regions (Fig. 3): Greater Accra, Volta, Central, and Western regions. For the study, eight communities within eight districts were selected, two from each region (Table 1). These districts were chosen due to their proximity to the coastline. The proximity of a fish source is an important determinant of household fish consumption behavior. Again, households in coastal areas are more likely to consume fresh or processed fish. Coastal communities are known for consuming small pelagic fish as a source of protein because they are affordable and readily available (FAO 2010).

**Table 1:** Number of households included in the study categorised according to region

Region	District and No. of Households	Total Households (Videotaped) (29)
Greater Accra	Ningo-Prampram (5) Krowor (5)	10
Central	Asebu/Gomoa (3) Mfantiman MA (3)	6
Western	Nzema East (3)	
	Shama (4)	7
Volta	Ketu South (3)	
	Anloga (3)	6

## Data collection

A semi-structured open-ended household questionnaire was used to collect primary data on demographics and the small fish species, including the parts that are removed and the reasons. In this study, data were gathered through face-to-face interviews, observation, and audio and video recorders. Prior to data collection, a pilot study was done to ensure its suitability and appropriateness, determining whether the questions were clear and understandable to the respondents. Before the interview, the research team established rapport with the community participants with the assistance of community leaders. Respondents were not told what day or time the research team would arrive. This was done to avoid biasing or influencing the cooking processes.

### Selection of participants

A multi-stage sampling process was employed to select the respondents. Communities in the coastal regions and fishing communities were purposely selected for this survey. First, stratified sampling was used to categorize the community’s housing units as poor, middle or rich. Poor households were defined as houses in slums or congested areas, families living in one room, houses in poor condition or located in poor environments, houses lacking proper waste management items such as garbage cans, and buildings that appeared to be crumbling (GPIR 2016). Only poor households based on our definition were sampled. Second, for the survey, households that cook and eat small fish were chosen using systematic and purposive sampling techniques. A distinguishable community landmark was identified and used as the starting point for household sampling (in most cases, the chief fisherman’s house). To ensure consistency, every household was selected from within 100–150 m of the north, south, east, and west of the observed landmark. In compound houses with more than one occupant, the person who occupied the first room was chosen for the survey unless the occupant was ineligible or unwilling. In such cases, the most willing household head in the compound was chosen. According to Thagaard (2009), the size of the sampling should be assessed to a saturation point. This implies that the sample is sufficiently large when studies of more units do not give a further understanding of the phenomenon. It is also a point that the number of informants or respondents in qualitative samples should not exceed the number that can be analyzed thoroughly. The survey lasted for 24 days and ended when the research team reached 102 households, at which point they were no longer obtaining new information. In total, 102 households were visited by the research team, but not all of them agreed to be interviewed. Out of the 102 households, 73 household members were interviewed but not videotaped. The reason for this was because either they were not cooking at the time of the visit or they declined to allow the research team to film the cooking process.

However, toward the end of the data collection, the research team convinced 29 respondents to allow them to videotape the cooking process in addition to the interviews. These 29 households were included in the analysis presented in the paper.

### Research instrument

The study used an interview guide (Supplementary material I) together with an audio and a video recorder as research instruments to collect information.

### Thematic data analysis

Audio and video recordings were transcribed, organized, and coded. The data collected were reviewed after every interview to focus on themes and patterns that emerged in subsequent interviews and observations. Data transcription involves listening to the tape and simultaneously noting down everything that was said on the tape (Mack et al. 2005). Data were analyzed after transcription using inductive analysis, concepts, themes, and models through interpretations made from the raw data (Thomas 2006). Open coding analysis was performed by naming and categorizing the data (Lawrence et al. 2013), followed by thematic network analysis, where codes are identified from textual data. The information obtained was clustered into basic themes, organizing themes, and finally a global theme (Attride-Stirling 2001) (Table 2).

**Table 2:** Codes, basic themes, and organized themes identified from textual analyses of transcription of audio and video recordings

*Codes*	*Basic themes*	*Organizing Themes*
*…the small fish gives a lot of blood*	*Nutritional value of small fish*	*Reasons for consuming small fish*
*…eating small fish gives me protein. …eating small fish makes my children grow well*		
*…. eating small fish makes bones stronger*		
*…. small fish is not expensive*	*Cost of small fish*	
*…. we get it for free*		
*…. Very cheap when bought in bulk*		
*……I get a lot for cheap*		
*……I do not need too much money*		
*……small fish is delicious*	*Good taste of small fish*	
*……. small fish tastes good*		
*……small fish is delightful*		
*……. small fish makes food taste nice*		
*……. small fish feels good in my mouth*		
*…. we ate small fish even as children*	*Consuming small fish as a tradition*	
*…. our parents gave us small fish*		
*…. we cannot eat without small fish at home*		
*…. small fish is our family food*		
*…. small fish known in our culture*		
*…..those parts cannot be eaten, it has a lot of bones*	*Unconsumable Small fish parts*	*Reasons for eating small fish whole or removing parts*
*…..the head bony*		
*…..the head contains stones*		
*…..those parts are not food*		
*…. the guts can spoil your food*	*Bad taste of small fish parts*	
*…. the guts taste bad*		
*…. the head has no good taste*		
*…. I do not like the taste of the guts and head*		
*…there is nice taste in the head of anchovy*	*Good taste of small fish*	
*…it tastes better eating all of it*		
*…it delicious when eaten whole with a local food*		
*…. every part taste good*		
*…the bones give calcium*	*Nutritional value of small fish*	
*…the nutrient in the whole fish makes us healthy*		
*….my children are smart because of the brain in the head*		
*…. eating the whole fish prevents diseases*		
*…. gives blood when eaten whole*		
*… you can eat it quickly*	*Preferring processed small fish*	*Preferring fresh or processed small fish*
*…. fresh Anchovies will break when boiled*		
*…. gives it a unique smell and taste*		
*….. takes away the excess water*		
*…I like to work on it by myself. …It is better when it is fresh*	*Preferring fresh small fish*	
*…it tastes amazing when fresh and spicy*		

### Verification and credibility

The findings of the research were disseminated to each community (households) before the final departure. At the end of each interview and observation, the major findings were shared with the respondents and confirmed by all participants of the data collection process. Primary data generation, in-depth engagement, and crosschecking the data with the communities through the individual respondents were all ensured to have credible and dependable research data.

## Results

### Participant demographics

Data from 29 videotaped households were analyzed in the study (Fig. 3). Ten (10) respondents in the Greater Accra, seven (7) respondents in the Western, and six (6) respondents each in the Central and the Volta regions of Ghana were interviewed and videotaped (observation) as they prepared and cooked a meal containing small fish (Table 1).

The inquiry commenced with questions about the respondent’s household and an idea of their economic status (occupation), and if they preferred small fish. The respondents were within the age bracket 30–50 years, with a married majority and only two men. The average household size was five persons, and nearly all the respondents had a low educational level, with all living in a fishing community. Only two of the respondents were unemployed, with the majority (12) being fish processors only, and eight were fish traders in addition to processing fish (Table 3).

**Table 3:** Characteristics of respondents

Variable	Frequency (n)	Percent (%)
Sex		
Female	27	93.1
Male	2	6.9
Occupation		
Cobbler	1	3.4
Fish processor	12	41.4
Fish trader	3	10.3
Fish trader/processor	8	27.6
Fisherman	1	3.4
Seamstress	1	3.4
Shop Attendant	1	3.4
Unemployed	2	6.9
Education		
JHS	10	34.5
Non-formal	13	44.8
Primary	6	20.7

*HH* Household, *JHS* Junior High School, *Non-formal* Education outside the formal classroom

### Food consumption and Household food utilization

Various small fish are consumed along the coastal regions of Ghana (Table 4); however, the most preferred ones are anchovies and herrings. Small fish are consumed in these fishing communities mostly based on their nutritional value, cost, good taste, and because of tradition in no particular order. Most married as well as unemployed respondents who had completed Junior High School stated tradition and low cost as the main reasons behind their consumption. Married respondents who are female fish traders and processors with non-formal education said that their consumption of small fish is based on nutrition and taste. Nevertheless, during data analysis, these demographic characteristics had no significant impact on the reasons for the preference for small fish as narrated by the various categories of respondents, as their answers were comparable throughout the data collection period.

**Table 4:** The predominant small fish species consumed

Common name	Scientific name	Local name	Habitat	Times mentioned	Times cited as most liked
Herring	*Sardinella*	Amane/emane^2^	Marine, pelagic	17	15
Anchovy	*Engraulis encrasicolus*	Abobi^1^	Marine, pelagic	20	12
Lesser African threadfin	*Galeoides decadactylus*	Sukue^1^	Marine, demersal	2	1
Congo dentex/ red pandora	*Pagellus bellottii*	Yiyiwa^1^	Marine, demersal	1	1
*Other Species	*_*	_	_	15	0

^1^Local names from Kwei and Ofori-Adu (2005)

^2^Online twi dictionary (www.mytwidictionary.com)

^*^Other species: African moonfish, big eye grunt, West African pygmy herring, cassava croaker, sharp chin flying fish, barracuda, red mullet, sea tilapia

Though responses were given regarding the less commonly consumed small fish species, these are not presented in detail since the majority of the accounts were dominated by anchovies and herrings; and because it answers the research question on the commonly consumed small fish.

### Household food utilization (food preferences and food preparation)

Pertaining to the parts removed from the small fish during preparation, the respondents gave various reasons for taking out those parts. Whether a small fish was eaten whole or some parts removed depended on the species.

#### Anchovy

From the study, it was observed that anchovies were preferred whole with all body parts intact (i.e., with the head, tissue, viscera, and bones) by all respondents, and only one respondent removed the viscera during preparation. It was observed that, regardless of the processing method used, the respondents preferred to consume small fish with all their parts intact. The participants cited two main reasons for this preference. Firstly, they believed that the bones contain important nutrients such as calcium, which are beneficial for their health and that of their children. Secondly, they stated that they could not bear to discard any part of the fish due to its delicious taste.

All 20 (100%) respondents who mentioned anchovies as a preference indicated that they like already dried, smoked, or fried anchovies and do not cook them fresh. According to the data collected, this is because the small fish in the processed form taste delicious, are nutritious, affordable, and easy to consume.

#### Herring

All 17 (100%) respondents who mentioned herring as one of their most preferred small fish species removed some parts during meal preparation and consumption. When the herring is processed, i.e., smoked, it is done with all body parts intact, including the head and viscera; however, during our observations, we noticed that both the head and viscera were discarded when the small fish was used in soup or in meal preparation. Our respondents’ explanations for their actions were that the head is bony and the viscera taste bitter and would therefore spoil their meal.

During the data collection, the remaining 47% of respondents who mentioned herring as one of their most preferred small fish species indicated that they like fresh herring, with their main reason being that “*it tastes amazing when fresh and spicy.”* Based on the observations and narrations from the respondents, we found that when preparing fresh herring, the usual practice is to remove the guts, fins, gills, and scales before cooking. Additionally, the head is typically not consumed due to the presence of sand. The respondents also perceive these discarded parts, such as the head and fins, to be less nutritious compared to the rest of the fish. For preparations that include the head, i.e., when the fish is to be smoked, only the bony parts of the fish’s mouth are cut off, while the head is cut off for the herring that is to be steamed or boiled. They then go on to remove the fins and scale the fish on both sides for both preparations. When the fish is to be steamed, the gills are also removed, then it is cut open to remove the viscera; the tail is usually the last part to be removed. All the removed unwanted parts are dropped in the same bowl containing the remaining fish parts. The wanted parts are then put in another bowl containing clean water and washed before being used for their purpose, such as boiling or steaming.

Fifty-three percent (9 of 17) of the respondents who mentioned herring as one of their most preferred small fish species wanted it in the processed form, specifically when smoked. This form was preferred because, according to the respondents, it tastes and smells better, and it is more delicious when the excess water is drained through the smoking process.

## Discussion

The most preferred small fish species identified in this study were anchovies and herrings. According to Nunoo et al. (2015) and Ashitey and Flake (2009), these species are among the most important species in Ghana. Anchovies were preferred fried, dried, or smoked and were eaten whole, including the head. Herrings, on the other hand, were preferred either fresh or smoked. Fresh herrings were boiled after removing the head, gills, fins, tails, and viscera. Herrings were smoked with the head and viscera, but both were removed before being added to boiling soup. These are important findings as both processing methods and the parts of the fish included in a meal will impact the nutrient composition. Analyses of whole fish samples of sardine and anchovies, compared to whole fish samples of the same species where head, fins and viscera are removed, showed that removing these parts results in lower content of vitamin A, folic acid, iodine, calcium, iron and zinc (Aakre et al. 2020). The respondents reported various reasons for their choices, e.g., keeping nutrient content or removing parts that taste bad or contain stones.

The preparation practice observed in this study was similar to what Roos (2001) reported among rural Bangladesh women – the women were asked to clean sampled fish as they normally did – the parts that were removed included the gill cover, jaw, head, fin, tail and/or viscera. The answers show that the consumers have clear ideas about nutrition, albeit in a quite instrumental manner (eating bones strengthens the skeleton; eating brain strengthens the brain).

Aside from the nutritional aspect, there could be a risk of contamination (food safety) due to improper preparation practices used by the respondents in the study at a certain point of the observation. For instance, releasing the visceral of the fish into the same bowl containing the edible fish parts. Feglo (2004) mentioned that improper preparation and hygiene practices could increase the likelihood of the growth of microorganisms, including pathogenic bacteria from fecal sources. Though these kinds of studies in Ghana may be limited, Hasselberg (2020) reported acceptable levels of total cell counts but high coliform bacteria count in processed fish samples, which might indicate fecal contamination.

The study showed that small fish was prepared using traditional methods such as sun drying, smoking, and frying because they are cheap. Cooking of fresh herrings was preferred by the fishermen. Several responses from the study indicated that herrings are preferred smoked. Nunoo (2015) explained in his study that the main species that are smoked traditionally included anchovies and herrings; however, a similar significant number of the respondents (8 of 17) in this study liked fresh herrings, contrary to the findings of Nunoo et al. (2015). A notable difference was that the respondents who usually preferred fresh herrings mostly had access to fresh fish through partners who worked as fishermen or had direct contact with the fishermen, so they did not stress the drying, which can take 3 days, while respondents who had no direct contact with fishermen preferred the smoked or sundried form of the fish. The difference could therefore have been the target population used in each of the studies and the respondent’s location and direct access to fishermen.

The effects of sun drying and smoking on the nutritional content of small fish have been investigated in a limited number of studies. Roos et al. (2002) found that sun drying retains the nutritional value for protein, fat and minerals (iron, zinc, and calcium) in small fish but destroys all vitamin A. In a recent market study from Ghana, most of the smoked fish analyzed had low vitamin A content despite being whole fish samples containing the head and viscera (Hasselberg et al. 2020). Thus, analytical data on processed fish and prepared meals considering what parts are eaten are needed to calculate nutrient intake from fish consumption. However, this information is also needed to understand where in the fish value chain nutrients are lost.

## Conclusion

In the present work, we have addressed “Food Consumption” and “Household Food Utilization” within the IPC Integrated Food Security and Nutrition Conceptual Framework. The food preferences were influenced by food access, i.e., proximity to a fisherman, taste, and nutritional quality, while demographic characteristics did not affect small fish preferences. The respondents preferred anchovies and herring, but a range of small fish were consumed. Anchovies were consumed whole with the head and all other body parts intact regardless of processing and preparation method. For herring, the preparation method depended on access to fresh fish. Herring was preferred in the fresh form, and when fresh, the head and viscera were discarded before cooking. Smoked herring was consumed whole with the head, viscera, and other parts intact. Nutrient content depends on species, processing method and tissue consumed; thus, these results will be of importance for further studies into small fish preparation and processing, as well as sampling schemes for food composition tables and for calculation of nutrient intake from small fish in the future.

## Supplementary Information

Below is the link to the electronic supplementary material.

Supplementary file1 (DOCX 694 KB)

## Data Availability

The findings of the research were disseminated to each community (households) before the final departure in which approximately 29 respondents participated. At the end of each interview and observation, the major findings were shared with the respondents and confirmed by all participants of the data collection process.
