# Supplementary figures and images for: The processing, preparation, and cooking practices of small fish among poor Ghanaian households: An exploratory qualitative study
Source: Marit Stud. 2023 Apr 12;22(2):15. doi: 10.1007/s40152-023-00300-w (PMC10092916; doi:10.1007/s40152-023-00300-w)

1. **
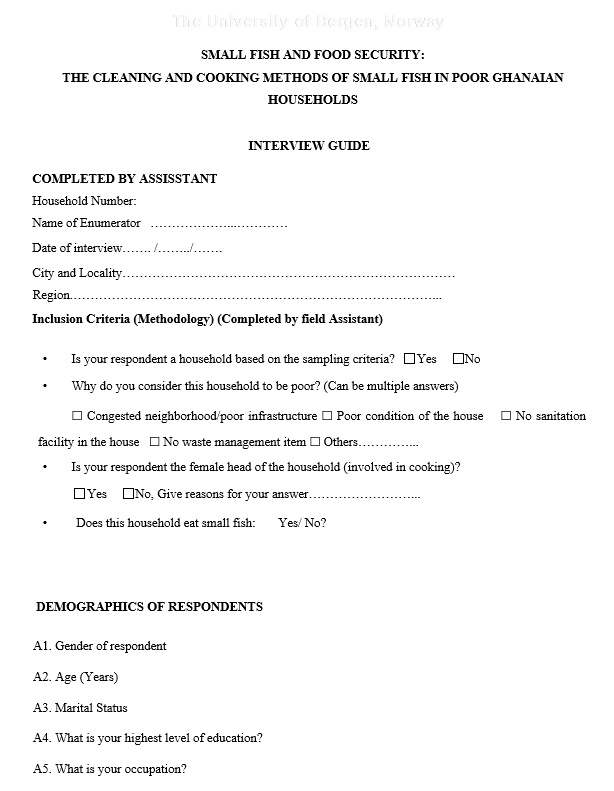
Interview Guide**

**
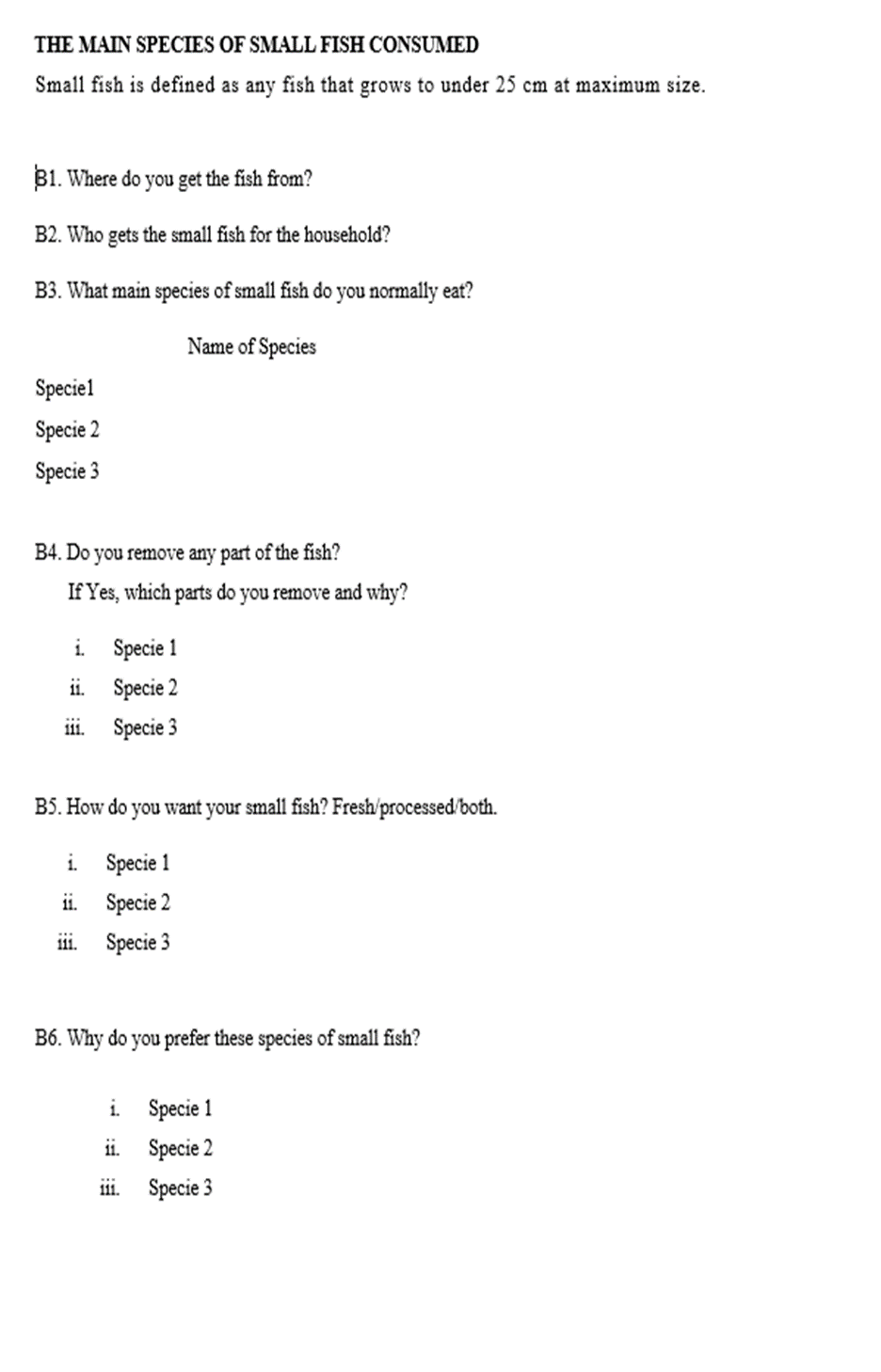
**

**
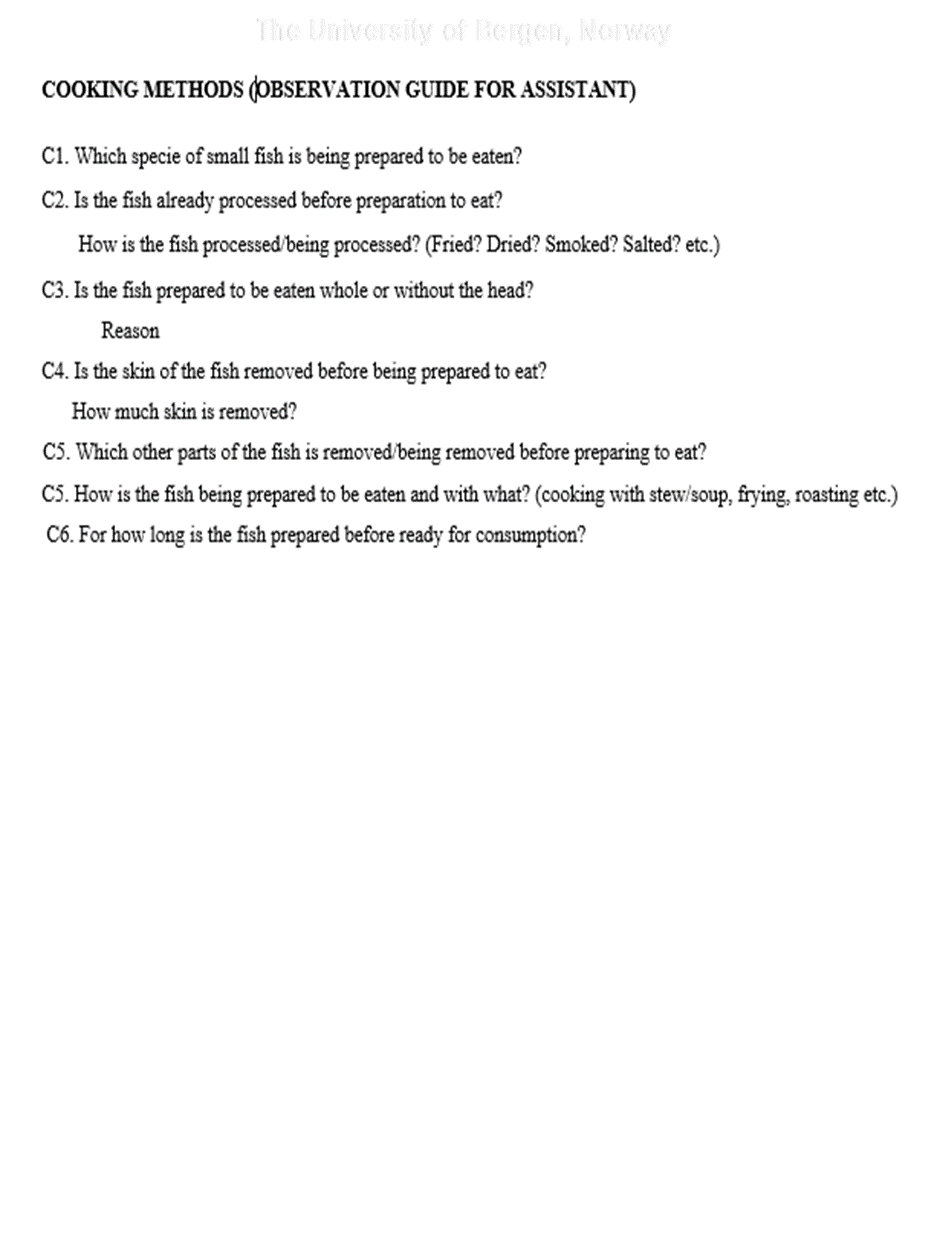
**

1.
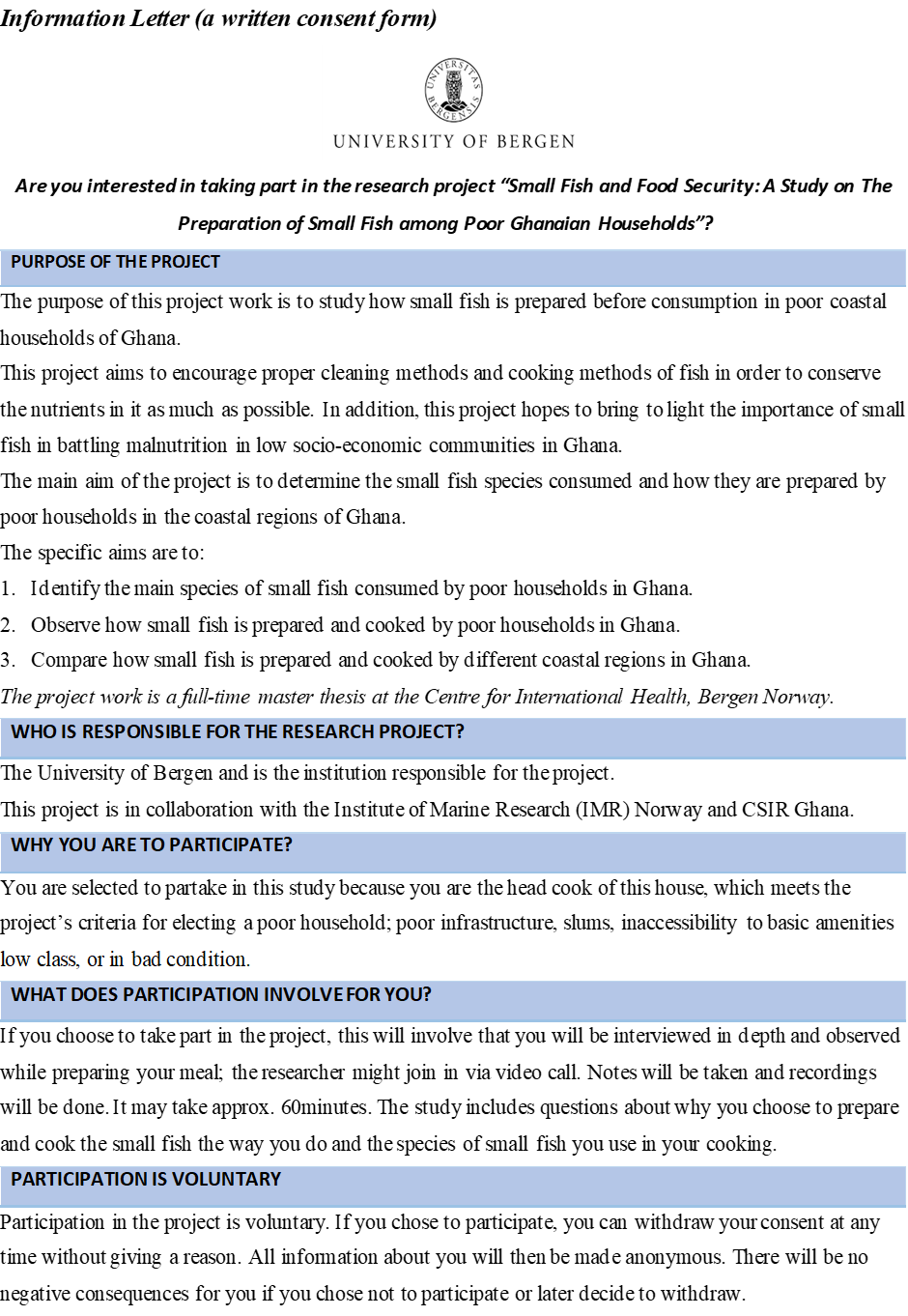
**Information Letter**


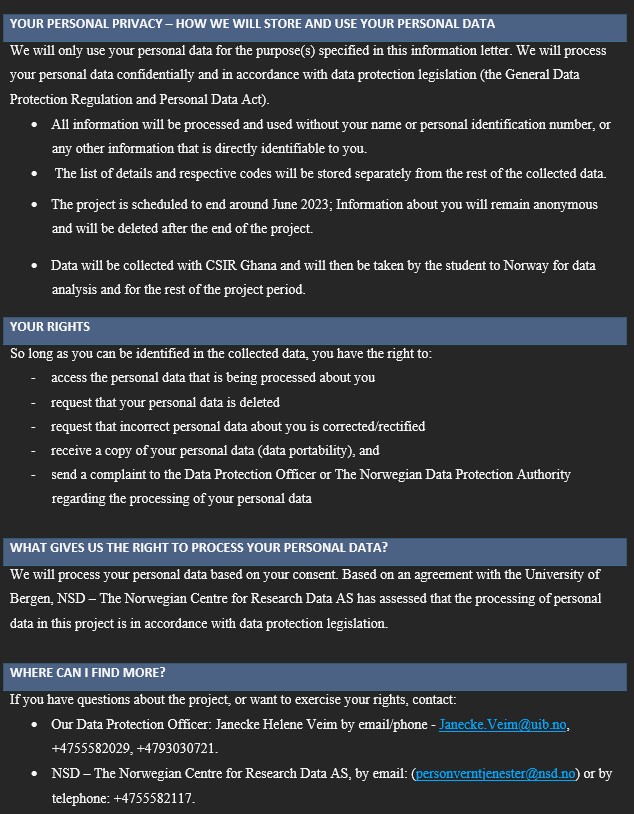


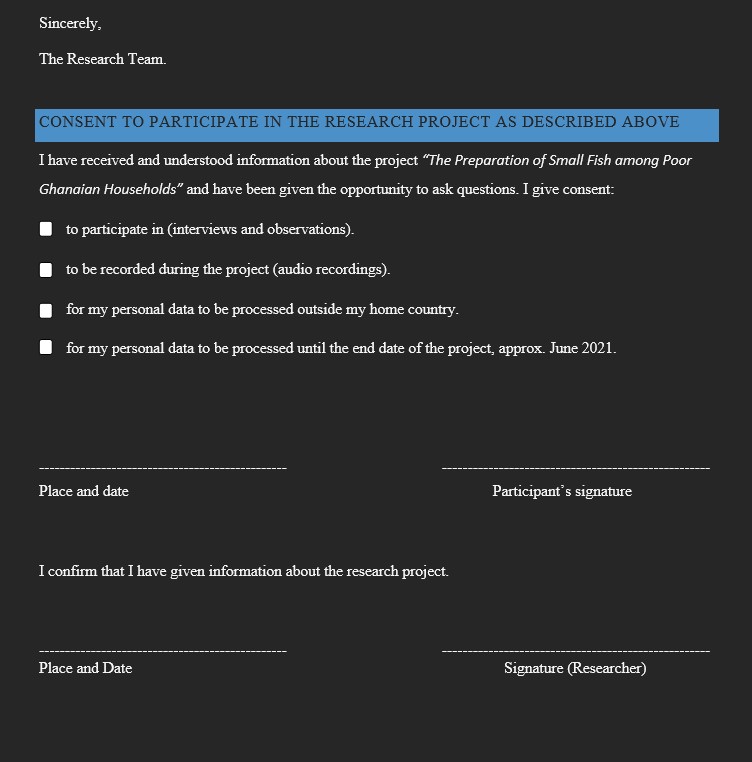

Supplement: Supplementary file 1 — Supplementary file1 (DOCX 694 KB) [file 40152_2023_300_MOESM1_ESM.docx]
